# Eating Disorders and Disordered Eating in Competitive Cycling: A Scoping Review

**DOI:** 10.3390/bs12120490

**Published:** 2022-12-02

**Authors:** Charlie Jon Roberts, Howard Thomas Hurst, Jack Hardwicke

**Affiliations:** 1 Centre for Physical Activity and Life Sciences, University of Northampton, Northampton NN1 5PH, UK; 2 Centre for Applied Sport Physical Activity and Performance, University of Central Lancashire, Preston PR1 2HE, UK

**Keywords:** eating disorders, disordered eating, competitive cycling, eating behaviours

## Abstract

This article reports on the findings from a scoping review on eating disorders and disordered eating in competitive cycling. The review was informed by a scoping review methodological framework as well as the Preferred Reporting Items for Systematic Reviews and Meta-analysis extension for scoping reviews (PRISMA-ScR) reporting guidelines. PubMed, SPORTDiscus and Web of Science were used to identify relevant literature for review. Fourteen studies met the eligibility criteria and were included in the full review. A narrative synthesis was used to summarise the main findings and themes across the included literature. Findings from the review are presented under the following themes: cycling as an ‘at-risk’ discipline; power to weight ratio; energy requirements and risk of low energy availability; the social environment of cycling; nutrition support provision; relationship between eating disorders/disordered eating and exercise addiction; and recommendations made in identified literature. Overall, the literature suggests competitive cycling is a sport with a high prevalence of disordered eating and/or eating disorders and a sport with unique risk factors that contribute to this. Crucially, more research is needed in this area. The article concludes with the gaps in the literature highlighted, implications for future research, and applications to policy and practice suggested.

## 1. Introduction

Eating disorders (ED), such as anorexia nervosa, bulimia nervosa, binge eating, and eating disorders not otherwise specified, are characterised as severe psychiatric disturbances that cause persistent disruption to eating behaviours and body image perception resulting in significant impairment to physiological health and psychosocial functioning [1]. Disordered eating (DE) behaviours such as skipping meals, restricting overall energy or macronutrient-specific food intake and purging can range in severity and may predict the onset of clinical ED development [2]. Globally, EDs present a major public health burden with high prevalence across the lifespan [3]. The individual may experience comorbidity with other mental health conditions, physiological deterioration resulting in disorders such as osteoporosis, cachexia, and kidney disease [4], and increased risk of mortality, whilst additional burden is placed on healthcare systems [5].

Athletic populations have unique dietary requirements to account for increased energy expenditure in response to competition and training load. Furthermore, nutrient requirements to optimise health and performance and enhance recovery and adaptation will be increased but variable due to the demands of the athletic discipline and body composition goals [6]. DE behaviours present a spectrum for athletes; practices such as energy and macronutrient restriction may range from optimisation for competition or adaptation to being detrimental to health and characterised as a clinical ED [7]. Sport-specific risk factors include participating in aesthetic sports and/or sports whereby low overall and/or fat mass is advantageous, performance pressure and engaging in team weigh-ins [8]. Furthermore, reports suggest ED prevalence may be higher in athletes than the general population [9]. However, true rates are difficult to quantify; the negative stigma surrounding EDs may result in individuals feeling reluctant to disclose and seek help regarding their behaviours and potential condition [10]. This phenomenon also poses methodological and ethical issues for researchers in the area, making valid insights into the true prevalence rates of EDs among athletic populations difficult to ascertain.

Competitive cycling encompasses a range of disciplines (e.g., road, track, BMX, cyclo-cross and mountain biking) that present variable individual, training and competition demands [11]. Concerns for DE have been raised across cycle disciplines, and specifically in road-based disciplines [12]. This is likely due to the specific demands of road-based disciplines. For example, road cycling is an extreme, endurance-based sport [13] with the approximate figure of kilometers covered by professional road cyclists per year in training and competition being between 25,000–35,000 km [14]. Additionally, for cyclists specialising in climbing, the desire to maintain a high power to weight ratio may further contribute to DE. The large training volumes and competition demands associated with many cycle-sport disciplines result in substantial energy expenditure that are often difficult to compensate for via dietary intake which may result in low energy availability [15].

Furthermore, it has been reported that cyclists often perceive leanness to be important for success [16] and can thus manipulate body composition accordingly [11,17]. As such, competitive cyclists may be uniquely vulnerable to engaging in DE behaviours and developing clinical EDs. The social environment of competitive cycling may further exacerbate DE, with a desire for peak performance and emphasis on leanness encouraging athletes to engage in potentially harmful eating behaviours that can ultimately result in fatigue, injury, or illness [17]. Within the cycling community, anecdotal reports suggest that DE is an issue at both professional and amateur levels of the sport, particularly in road cycling [18,19]. However, there has been limited academic attention to this issue and no synthesis of the existing literature on the topic. As such, the purpose of this scoping review is to assess and discuss the current literature concerning EDs and/or DE patterns in competitive cyclists, identify evidence gaps and suggest directions for future research and practice.

## 2. Materials and Methods

### 2.1. Methodological Rationale

With the paucity of literature in the area of interest and diversity in study design, population groups and measurement instruments, it was deemed that a scoping review with a qualitative narrative synthesis of findings was more appropriate than a systematic review. The purpose of a scoping review is to determine the current state of research in a specific field [20]. It is considered an appropriate method when the field of interest is diverse in terms of study methods and/or when there has not been a prior synthesis of research on the topic. With this paper reporting on the first review into disordered eating and eating disorders in competitive cycling, a further rationale for the approach is highlighted.

As such, a scoping review was undertaken, and the five-stage process methodological framework outlined by Arksey and O’Malley [20] was followed. Additionally, the Preferred Reporting Items for Systematic Reviews and Meta-analysis extension for scoping reviews (PRISMA-ScR) reporting guidelines was used to direct the protocol followed.

### 2.2. Stage 1: Identifying the Research Question

After a preliminary search of the literature suggesting limited research into the field of interest, the authors agreed the research question needed to be broad and exploratory in nature. As such, the research question for this scoping review was: What is the state of current evidence on EDs and DE in competitive cycling?

### 2.3. Stage 2: Identifying Relevant Studies

The literature search had no defined date parameters, with the review being undertaken in September 2022. The following databases were searched: PubMed, SPORTDiscus and Web of Science. The search strategy was decided amongst the authors with the agreed key terms detailed below. Boolean operators “AND” and “OR” were used to refine literature searches. Due to the limited literature in the area, the research team agreed on searching competitive cycling as a broad search term, rather than search for specific cycle sport disciplines. Specific cycle sport disciplines, where mentioned, were extracted as detailed below. Additional manual searches for literature were undertaken by reviewing the included studies reference lists. Google Scholar was also used to supplement the identification of relevant studies, with the same search terms used. The inclusion and exclusion criteria for identified studies can be seen below.

Key search terms for all databases:
“Disordered eating” “cyclists” “competitive cycling”
“Eating disorders” “cyclists” “competitive cycling”
“Eating patterns” “cyclists” “competitive cycling”
“Eating attitudes” “cyclists” “competitive cycling”
“Nutrition habits” “cyclists” “competitive cycling”
“Race weight” “cyclists” “competitive cycling”
“leanness” “cyclists” “competitive cycling”
“Eating disorders” “anorexia” “bulimia” “disordered eating” “cyclists” “competitive cycling”
Inclusion criteria were:
Articles written in English;
Peer-reviewed articles;
Articles that include specific reference to Eating Disorders and/or Disorder Eating in competitive cycling;
Articles related to the areas above with the addition of eating patterns, attitudes and nutrition habits in competitive cycling;
Articles with any reference to race weight and leanness;
Articles related to any of the above in any competitive cycling setting.
Exclusion criteria were:
If dietary or nutrient intake were measured with no reference to disordered eating patterns or eating disorders;
Articles not published;
Articles without full-text access;
Grey literature;
Articles not written in English.

### 2.4. Stage 3: Study Selection

**Figure 1:**
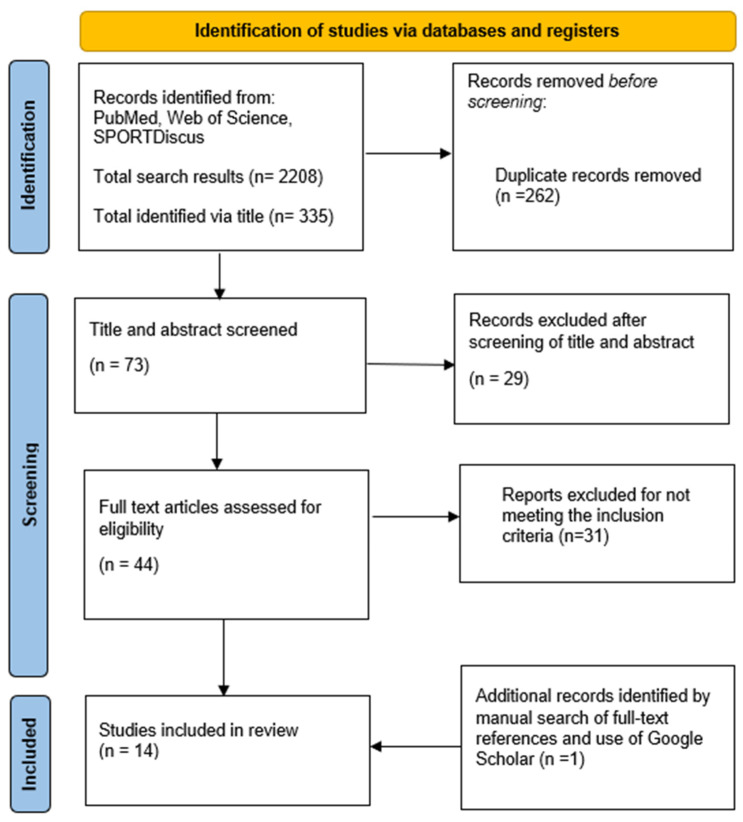
Flow diagram of search process.

Studies returned from the above search strategy were subject to a 3-phase review against the eligibility criteria. First, CJR and JH reviewed all returned titles against the eligibility criteria and collated all relevant titles. Second, studies were then subject to a more detailed review of abstracts and sorted into ‘include’, ‘exclude’ or ‘uncertain’ groups. The final phase involved detailed readings of all ‘included’ and ‘uncertain’ studies, with full contents screened against the eligibility and inclusion criteria. Studies identified through supplementary search strategies (Google Scholar and reference list searching of included studies) were subjected to the same 3-phase review. Corresponding authors of studies with samples of mixed-sport athletes but not presenting results broken down by sport were contacted to request access to the data stratified by sport (eight corresponding authors were contacted with a request for data and none were returned). Total search returns were recorded from initial searches, with all discarded studies having reasons for exclusion documented. Figure 1 details the PRISMA flow diagram of study selection providing more detail on this process. CJR and JH conducted the literature search and any disagreements on study inclusion were discussed. HTH acted as a third reviewer if required.

### 2.5. Stage 4: Charting the Data

Once the studies eligible for full review were established, CJR and JH extracted the desired data from each included study. HTH reviewed all data extraction against the original studies to assess and ensure accuracy of extraction and synthesis. The categories included for data extraction were informed by recommendations outlined by the framework followed [20], as well as more specific categories relevant for this review which were agreed amongst the research team. Specifically, the following was extracted from the eligible studies:

Author;

Year of publication;

Journal/Source of publication;

Geographic location of study;

Aims/Purpose of the study;

Study population and sample characteristics;

Cycling discipline;

Study design;

Measurement instrument and details of these (if applicable);

Key findings and recommendations;

Limitations;

Any important additional notes that relate to the scoping review research question outlined above.

### 2.6. Stage 5: Collating, Summarising and Reporting the Results

The results were collated, summarised, and reported to provide a detailed overview and summary of existing research on DE and EDs in competitive cycling. A narrative synthesis was used to summarise the main findings. Specifically, we first describe the general characteristics and findings of the included studies and then present a thematic summary of the findings across the included studies. In accordance with guidelines for scoping reviews, no assessment of risk of bias, quality assessment or meta-analysis were conducted for this scoping review [21].

## 3. Findings and Discussion

The flow of study selection is presented in Figure 1. Briefly, 14 studies were included in the final thematic summary following the removal of duplicates and full manuscript screening. Relevant data extracted from included studies are presented in Table 1.

Following full screening of the included studies, several themes were identified for further expansion and presentation of findings. The themes are: cycling as an ‘at-risk’ discipline; power to weight ratio; energy requirements and risk of low energy availability; the social environment of cycling; nutrition support provision; relationship between EDs/DE and exercise addiction; and recommendations made in identified literature.

### 3.1. Cycling as an ‘at-Risk’ Sport

Across the reviewed studies, an overarching theme was the suggestion of competitive cycling being an ‘at-risk’ sport for ED and/or DE with increased prevalence compared to other sports. Studies reported on in Table 1 suggested high prevalence rates in competitive cycling, particularly road cycling. However, it is hard to make claims with certainty due to the heterogeneity in methodologies and reporting of ED and/or DE across studies in the area making comparisons within the sport, and across sports, difficult. Further, it is likely there is under-reporting of ED and/or DE in competitive cycling due to sensitivities around the issue and social stigma [10]. Nonetheless, it was clear from the scoping review that competitive cycling presents as an ‘at-risk’ sport for having problems with ED and/or DE and has unique risk factors that contribute to this. What follows are more specific themes that were apparent across the studies that may explain this over-arching theme.

### 3.2. Power to Weight Ratio

It has been suggested that athletes competing in sports in which low body weight improves sports performance are at an increased risk for EDs [22]. This can be compounded by increased pressure and focus to attain what is perceived as a desirable body weight within such sports, as athletes attempt to gain competitive advantages over one another. Competitive cycling, particularly road-based disciplines, present as a sport in which strong emphasis is placed on the power to weight ratio of the athlete as a determinant of success [23]. Therefore, it has also been highlighted as a sport that may have unique risk factors for EDs and/or DE. The notion of power to weight ratio was recurrent across numerous reviewed studies as a potential influential factor that led to EDs and/or DE due to the focus on and preoccupation with body weight it necessitates.

Muros et al. [12] suggested that the high prevalence of ED found in their sample of Spanish cyclists may be explained by the watt per kilo ratio, and particularly the impact of this on performance in cycling. Both Hoon et al. [24] and Haakonssen et al. [25] reported cyclists were conscious about body weight due to its perceived relationship to performance. Cyclists in both studies reported the belief that a lower body weight was optimal for cycling performance and that they performed best when at low body weight. This led to cyclists across both studies reporting engaging in conscious attempts to manipulate body weight. This occurred on a continuum whereby at one extreme were practices such as purging and prolonged training periods without eating toward more controlled calorie reduction for performance benefit. Wells et al. [7] have highlighted that EDs in athletes occur on a spectrum from dietary changes for performance optimisation to eating behaviours that can negatively influence health and wellbeing. The papers reviewed did not explicitly report details as to the eating behaviours of cyclists regarding reduction in weight for performance, so inference is difficult.

However, it was clear that competitive cycling, specifically road cycling, is a weight-sensitive sport whereby athletes’ relationship to their body weight is influenced by perceptions of performance. This may lead to ED and/or DE through desires to manipulate body weight as a means to improve performance. This is particularly prevalent, and problematic, in sporting contexts where the environment is typically characterised by hyper-competition and a focus on performance, particularly at the elite levels of sport. As such, the specific characteristics and demands of competitive cycling, namely the focus on power to weight ratio, can be recognised as a risk factor for ED and/or DE.

### 3.3. Energy Requirements and Low Energy Availability

High energy expenditure is expected in competitive cyclists due to the demands of racing and training. An individual who cannot match the energy demands of exercise via dietary intake risks being in a low-energy available state, which can disrupt the body’s ability to maintain optimal physiological function and health, and compromise performance and recovery [26]. Whilst definitive guidelines for optimal energy availability (EA) currently do not exist, it is generally accepted that <30 and 45 kcal·kg^−1^ FFM·day^−1^ (FFM, fat free mass) is a threshold for balanced EA in males and females, respectively, beyond which both health and performance may be compromised [27,28]. Individuals may unintentionally consume less energy than is required to match exercise demands due to limited awareness of competition nutrition fueling strategies [29] restricted time, facilities, or skills for cooking appropriate meals [15] and financial barriers [30]. As such, low EA may be a complication of DE behaviours and must be considered due to potentially adverse health and performance outcomes [26].

Low EA disrupts bone re-modelling [31], cognitive function [32] and neuromuscular function, which may be detrimental to health, well-being, and exercise performance [29]. Endocrine dysfunction occurs impacting male and female reproductive systems, with downregulation occurring to conserve energy [29]. Such hormonal perturbations in response to low EA may also adversely influence metabolism, further disrupting energy homeostasis [15]. Four days of low EA at 15 kcal·kg^−1^ FFM·day^−1^ daily is sufficient to suppress leptin and insulin in exercising males [33], leading to reductions in energy expenditure [34].

Viner et al. [35] observed a large prevalence of low EA in male and female road cyclists and mountain bikers. Male athletes appeared to present lower EA than female athletes during the pre-season (male:18.8 ± 12.1; female: 26.2 ± 14.1 kcal·kg^−1^ FFM·day^−1^), competition (male: 19.5 ± 8.5; female: 25.5 ± 3.1 kcal·kg^−1^ FFM·day^−1^) and off-season (male: 21.7 ± 9.2; female: 23.8 ± 8.9 kcal·kg^−1^ FFM·day^−1^). Furthermore, road cyclists reported greater EA than mountain bikers at all points across a competitive season. An energy availability of 16 ± 18 kcal·kg^−1^ FFM-1·day^−1^ was reported in highly trained male competitive cyclists on training days. The authors detail average on- and off-bike training volumes of 18.4 h per week; however, no quantification of per-day volumes is presented [36]. Such studies should be interpreted with caution as energy intake and expenditure in the studies reviewed are estimated from self-reported measurements [37] and thus may mask the true prevalence of low EA in this population.

### 3.4. The Social Environment of Competitive Cycling

Sociocultural factors (e.g., peer pressure and media influence) have been reported as risk factors for ED and/or DE in athletic populations [9]. Through such factors, there can be sociological pressures for athletes to manipulate body composition to align with what is deemed optimal for their sport [38]. As noted above, competitive cycling is a sport in which the typical anthropometrics required to excel include low body weight and high-power output, leading to a desired body type characterised by low fat mass relative to lean mass. The awareness of this desired body type amongst competitive cyclists was clear, and the social environment of competitive cycling was reported among the included studies as an influential factor for eating disorders in the sport.

Hoon et al. [24] reported that 86% (n = 97) of respondents to their survey either agreed or strongly agreed that male cyclists are a weight-conscious population, and 69% reported a belief that being at their lowest-ever body weight was beneficial for cycling performance. Furthermore, 79% (n = 77) of the cyclists surveyed reported having deliberately tried to manipulate their body weight in the preceding 12 months, with 95% of these doing so with the aim to lower their weight. In an attempt to achieve the goal of weight reduction, participants reported a variety of weight loss strategies with the most common being a deliberate reduction in food intake (n = 69) and an avoidance of sugary foods (n = 55). A similar finding was reported by Haakonssen et al. [25] where 26 (70%) of the 37 cyclists surveyed reported attempting to reduce their body weight in the past 12 months. Five of the cyclists in this study reported having been previously diagnosed by a physician as having an ED. The authors of both studies conclude that the competitive cyclists studied were a weight- conscious population and that body weight manipulation is a known and accepted feature of competitive cycling culture.

Related to a consciousness around body weight was that of body image. De Bruin and Oudejans [39] highlighted that the cyclist interviewed in their mixed-sport sample reported an increased body weight awareness within the elite sport arena and thin-idealisation within the sporting context. This was perpetuated in the sport due to the tight fitting, and often revealing, sports clothing required to participate, a concern shared with rowing, athletics, gymnastics, and dance. This was reported to heighten the cyclist’s (and other athletes in the sample) body awareness, negative self-evaluations, and eating behaviours [39]. Filaire et al. [40] also reported body image-related issues in their sample of elite male judoists and cyclists. The self-reported survey data suggested the judo and cyclist athletes had significantly lower body-esteem appearance and weight satisfaction compared to the control group that did not engage with competitive sport.

With the social environment of competitive cycling seemingly influencing cyclists’ attitudes toward body weight, it was reported that pressure from immediate peer networks contributed to eating behaviours. For example, Filaire et al. [40] reported that 46% of the 15 cyclists in the study felt pressure to lose weight, with such pressure coming from coaches, teammates, or themselves. Further, De Bruin and Oudejans [39] highlighted that 12 cyclists (32%) reported that their coach or sports director had expressed dissatisfaction with their body weight at some point in the past 12 months. This was more commonly reported amongst professional cyclists. Ferrand and Brunet [41] reported that the social pressures experienced within the social environment of cycling can be a catalyst for encouraging cyclists to feel pressure to control their bodies. Sundgot-Borgen and Torstveit [38] have highlighted that weight-related pressure from coaches can be an influential factor on development of an eating disorder in athletes, including in cyclists. Amongst the reviewed studies, the role of coaches, teammates and team managers were all noted sources of pressure for these athletes to be hyper-aware of their body weight.

In summary, the review of the included studies suggests that the social environment a competitive cyclist occupies can influence the risk of ED and/or DE. Influential factors reported on included weight consciousness, body image norms and pressure from peer networks. These factors appear to be imbedded in the culture of the sport and provide an important area to understand when examining ED and/or DE in competitive cycling.

### 3.5. Nutrition Support

Nutrition practitioners are often viewed by athletes as trusted sources of nutrition information and as such, access can help facilitate positive dietary choices and behaviours [42] and practitioners are in a strategic position to support the monitoring and identification of DE behaviours or clinical EDs. Despite this, practitioner advice may be viewed negatively and ignored. De Bruin and Oudejans [39] report that cyclists are likely to reduce food intake volume to induce energy deficits without the guidance of nutrition practitioners, a practice that may increase the risk of macronutrient and/or micronutrient deficiency, under-fueling, and DE behaviours. Whilst the importance of providing support and advice towards reducing the risk of insufficient nutrient intake is highlighted [25] there are no specific recommendations for practitioners proposed in the literature relevant to the scoping review.

Nutrition knowledge is considered a modifiable factor that can potentially influence dietary habits, and greater levels of knowledge may be a facilitator of appropriate food choices for health and performance in athletes [43,44]. In a cohort of competitive male cyclists, sports nutrition knowledge as measured using the validated Sports Nutrition Knowledge Questionnaire was inversely associated with ED risk (r = −0.55, *p*= 0.006) [36]. Despite this, research in the general population may suggest the opposite: those with greater levels of nutrition knowledge may be more vulnerable to DE behaviours and body image concerns. A hypothesis is that those demonstrating such behaviours leads individuals to seek information regarding nutrition [45,46]. This highlights the multi-factorial issue associated with DE risk; practitioners are responsible for ensuring the individuals in their care possess the levels of knowledge required to feed themselves appropriately for health, well-being, performance, and recovery, but must be aware of potentially inadvertently enabling DE behaviours.

To summarise, access to nutrition support was addressed in few of the identified studies. Knowledge around appropriate dietary intake for health, well-being and performance is likely not sufficient [30]; as such, future research should address the availability and efficacy of sports nutrition practitioners within competitive cycling.

### 3.6. Relationship between Eating Disorders/Disordered Eating and Exercise Addiction

DE behaviours and exercise addiction do not exist in vacuums, with multiple factors similarly contributing to both, such as body dissatisfaction and a drive for thinness. The relationship between DE and exercise addiction is logical, with excessive exercise playing a role in the psychopathology of clinical EDs in athletes [47] and these behaviours being observed in individuals with such disorders [48,49]. Despite the rational link between the two, exercise addiction is currently not classified as a mental disorder [50] due in part to the limited evidence to establish clear diagnostic criteria and describe the course of such conditions [51]. Nonetheless, meta-analysis data in athletic and non-athletic adults suggest that exercise addiction is observed 3.5 times more in individuals with an ED, as defined by validated questionnaires, than those without [49].

Exercise addiction is difficult to identify, as exercise volume does not necessarily correspond to the presence of such behaviours [52]. A variety of questionnaires are used to quantify exercise addiction or dependence in the literature, such as, the 21-item Exercise Dependence Scale [53] and a single question on the 28-item Eating Disorder Examination Questionnaire [54]. However, as exercise addiction is not considered a mental disorder [50], no clinical diagnosis can be made.

Cyclists reported greater Eating Disorder Examination questionnaire scores than athletes in other disciplines in a mixed cohort, with 92% of the 24 cyclists likely to feel guilt when not exercising [55]. Conversely, cyclists with secondary exercise dependence, whereby dependence is secondary to facilitate psychiatric conditions such as EDs, demonstrated low exercise dependence scores. Using the Exercise Dependence Scale drive for thinness sub-scale and a potential score of 126.00 with lower scores indicating less exercise dependence symptoms, cyclists who were at-risk, symptomatic and asymptomatic of secondary exercise dependence rated 17.00, 12.06 and 15.00, respectively [56].

Collectively, conclusions regarding the relationship between EDs or DE and exercise addiction or dependence cannot be made due to the ambiguous and self-reported nature of self-reported questionnaires used to quantify such behaviours. However, it is highlighted as an area for future research.

### 3.7. Recommendations from Identified Literature

Various recommendations for future research and practical application are proposed in the identified literature. Of the identified studies, only one did not employ a self-reported cross-sectional measure of DE patterns. Qualitative research [41] and studies exploring the impact of DE on physiological outputs [57] are recommended to greater understand the impact of these behaviours on physical and mental health, well-being, and performance.

A recurring recommendation in the identified literature is the provision of education to athletes [24,36,58] and support staff [24,25,41] to ensure appropriate support is provided for identifying and assisting athletes suffering from EDs or DE behaviours. Such education includes providing resources on energy availability and to facilitate appropriate dietary intake [36] and improving the ability of athletes and support staff to identify DE behaviours [41,58], which is likely beneficial for early prevention and treatment of clinical EDs. Additionally, greater education provision may enable support staff to be sensitive to DE and not promote an environment where such behaviours can be exacerbated [25].

Another recommendation is that clinical diagnosis of EDs is required to better understand the risk factors [12] and perhaps allow for effective prevention and treatment strategies to be implemented in athletic populations. As mentioned, most of the identified literature utilised self-reported questionnaires to estimate DE and as such does not reflect the prevalence of clinical EDs or DE behaviours.

## 4. Conclusions and Recommendations

In conclusion, the current literature suggests competitive cyclists may be at-risk for exhibiting DE behaviours and/or clinical EDs. The sport presents unique risk factors that contribute to this and must be considered by those working or competing in the field. Prevalence rates of EDs in cyclists are difficult to quantify in the literature due to methodological issues and heterogeneity in outcome measures regarding diagnosis; however, data suggest that clinical and subclinical ED prevalence in endurance athletes (female: 24%; male: 9%) is greater than those in technical (female: 17%; male: 4%) or ballgame sports (female: 16%; male: 5%) [59]. The above recommendations outlined from the literature are echoed by the authors and will not be repeated here. However, based on the findings from the scoping review, the authors provide the following novel recommendations for researchers, stakeholders, and practitioners interested in EDs and DE in competitive cycling:

More research on the area is required, particularly using different methodologies. Of the 14 identified studies, one reported on qualitative findings from a single elite female cyclist regarding the role of body image perceptions in EDs [39]. The emotional terrain of this research area requires further qualitative research to highlight the experiences and meanings attached to such behaviours by athletes, as well as moving away from cross-sectional approaches to a behaviour that may be transient in its presentation in an individual. The current reliance on cross-sectional self-reported surveys using scaled measures is also limited in providing insight to the interpersonal experience of eating behaviours, which is crucial for practitioners to understand.

Greater detail and consistency is required in the literature when reporting the anthropometric and demographic information of participants, cycling discipline, levels and quantification of training and racing volume. Cycling discipline and level of competition were particularly problematic, with many studies not clearly stating the specific characteristics of the sample used. As displayed in Table 1, this heterogeneity across studies in reporting makes broader insights difficult to ascertain.

Greater attention to the social context of competitive cycling and how this can influence eating behaviours is required. Most studies mentioned socio-cultural factors as influential on eating behaviours in competitive cycling, but none held these as the main research focus. Future research should closer examine the social environment of competitive cycling and its influence on eating behaviours. For example, studies focused on the media as a source of (re)production of such behaviours would be a good departure point for future research.

**Table 1:** Overview of data extracted from reviewed studies.

Year Location	Sample Number (Sex)	Age (Years)	Body Mass	Height	Discipline	Level	Reported Cycling Hours	Study Aims	Study Design	Measurement Instrument	Findings Relevant to Scoping Review	Reported Limitations in Paper
Ferrand and Brunet 2004 [41] France	42 (M)	21.8 ± 3.7	Only BMI reported. 20.5 ± 1.4 kg/m^2^	Not reported	Not reported	Amateur classified as Regional (n = 12), National (n = 13) and elite (n = 17)	Not reported	To examine the associations between dimensions of perfectionism and eating disorder symptoms among 42 young male amateur cyclists (M = 21.8 yr., SD = 3.7) over the three performance categories (Elite, National, Regional).	Cross-sectional self-reported survey	Eating Attitudes Test (EAT-26); Multidimensional Perfectionism Scale	24 of the 42 participants scored above 20 on the EAT-26 *	The EAT-26 alone does not yield a specific diagnosis of an eating disorder.
Riebl et al., 2007 [58] USA	124 (61 cyclists and 63 non cyclists as control) (M)	31.6 ± 10.4 (cyclists) and 23 ± 6.3 (control)	72.5 ± 7.6 (cyclists) and 80.5 ± 16.1 kg (control)	Not reported	Not reported	Not reported	Inclusion criteria included training a minimum of 5 h on a bicycle each week throughout the year; have participated in cycling for no less than 1 year.	To determine the prevalence of subclinical disordered eating behaviours among male cyclists, whether male cyclists self-report having an eating disorder, and whether male cyclists meet the daily recommendations for the major food groups according to the Dietary Guidelines for Americans.	Cross-sectional self-reported survey	The Eating Attitudes Test-26 (EAT-26) (7–9), Survey of Eating Disorders Among Cyclists (SEDAC) (10), and a nutrition questionnaire were completed by the study participants.	12 of the participants scored above 20 on the EAT-26 * Five reported having an ED	The legitimacy of the responses from the nutrition questionnaire was specific to this study and was not validated. The format of the questionnaire may have caused some of the study participants to underreport their intake. Oils and discretionary energy were not included, and the narrow list of foods provided composing each food group may have caused the cyclists to not meet the energy requirements and daily servings suggested by the Dietary Guidelines for Americans. A more specific understanding of cyclists’ nutrition habits would result from direct comparison to a non-cycling control population.
Gorrell et al., 2019 [55] USA	612 mixed sport (24 cyclists) (M)	18–26 (M = 20.99)	Not reported	Not reported	Not reported	Not reported. Sample from National College Athletics Association (NCAA) schools	Not reported	To characterise unhealthy exercise and eating behaviour according to competitive athlete status, as well as per sport type.	Cross-sectional self-reported survey	Eating disorder examination questionnaire EDE-Q	17% of cyclists reported EDE-Q scores within a clinical range	Findings should also be interpreted with caution, as they may also be considerably underpowered for the sports with small sample sizes (n = 8). Furthermore, 42 individuals participated in a secondary competitive sport, but it is possible that participation in one sport led to sport-specific body dissatisfaction differing from the body ideal of a secondary sport; future work may investigate these individuals separately. These analyses were secondary, and cross-sectional; therefore, findings cannot comment on the temporality of the development of these eating and exercise behaviours. Finally, measures were self-reported, which may reveal less information than if participants were queried in a clinical interview.
Muros et al., 2020 [12] Spain	4037 cyclists and triathletes (cyclists = 2037) (F and M)	37.72 ± 9.67	Only BMI reported 23.74 ± 2.69 kg/m^2^	Not reported	Not reported	Not reported. Federated cyclists in Spain	10.94 ± 4.64	To describe and predict eating disorders according to sex, body mass index, age and sport discipline within a sample of athletes.	Cross-sectional self-reported survey	The revised restraint scale (RRS); the five-item SCOFF; the Mediterranean diet (MD) adherence screener (MEDAS)	19.8% cyclists reported SCOFF score ≥ 2, positively indicating DE behaviour	The main limitation of the present study is its cross-sectional design as this cannot establish casual relationships. The results must be interpreted with caution because the study only had self-reported measurements. Although the sample was large, self-selection bias means that generalizability is limited. Further, the present study only assessed prevalence of ED via a self-reported questionnaire. Although SCOFF is a widely used screening tool with good sensitivity and specificity, it can only discern individuals at greater likelihood of developing ED, and complete diagnosis requires clinical follow-up.
Filaire et al., 2007 [40] France	15 cyclists (44 full sample) (M)	21.2 ± 2.8	68.0 ± 6.5 kg	180 ± 0.06 cm	Road cycling	National	Weekly distance covered ranged between 600 and 750 km. The athletes took part in races each weekend, with distance ranging from 100 to 150 km.	To test the hypothesis that male athletes who feel pressured to maintain a specific body weight present an elevated risk of subclinical eating disorders.	Cross-sectional self-reported survey	Eating Attitudes Test (EAT-26); Multidimensional Perfectionism Scale; Body Esteem Scale; Profile of Mood States	46% of cyclists reported feeling pressured to lose weight, and 41% and 10% of cyclists reported using fasting and laxatives as a weight loss method, respectively	First, as with any study using self-reported measures, findings may be susceptible to selective or erroneous reporting. Self- reported measures always carry risks, especially with athletes who might not be forthcoming in their answers for fear of being eliminated from the team if they appear to be eating-disordered. Second, the EAT-26 alone does not yield a specific diagnosis of an eating disorder. However, the use of a self-administered questionnaire, which incorporates Diagnostic and Statistical Manual of Mental Disorders as in our study, increases the advantages of using this questionnaire, which has been used in many epidemiological or screening studies, even if each subscale should be interpreted independently. Finally, although the number of participants was small, there have been very few studies of eating attitudes in male athletes, since this is a relatively under-recognised problem.
Yates et al., 2003 [60] Not reported	190 (36 cyclists) (F and M)	Not reported	Not reported	Not reported	Not reported	Not reported	Not reported	To differentiate groups of highly conditioned, competitive athletes on the basis of Exercise Orientation Questionnaire (EOQ) scores and self-reported psychiatric symptoms.	Cross-sectional self-reported survey	Exercise Orientation Questionnaire (EOQ)	14% of cyclists scored above 3.67 threshold established for ED patients on 4-item self-loathing subscale from EOQ	Study was based on relatively small samples of athletes who volunteered, often in the context of a sport’s club. Larger, more diverse, samples could yield different results. Other athletic groups could be included, especially those with known high and low rates of ED symptoms. Symptom self-reports are subjective and may contain retrospective and other distortions. Further study should include semi-structured interviews to confirm and further describe problems or disorders.
Haakonssen et al., 2015 [25] Australia	37 (F)	18–36	58.4 ± 5.9 kg	170 ± 7 cm	Road cycling	Professional and amateur	Not reported	To investigate the satisfaction of elite female cyclists with their body weight (BW) in the context of race performance, the magnitude of BW manipulation, and the association of these variables with menstrual function.	Cross-sectional self-reported survey	Female Cyclist Weight Management Questionnaire (Created by research team)	Five cyclists (14%) reported having previously diagnosed ED Energy and macronutrient restriction, skipping meals, training without eating, purging, wearing additional clothes and plastic wraps whilst training and taking supplements or medication identified as body mass reduction techniques	None reported by authors
Hoon et al., 2019 [24] Australia	97 (M)	32.0 ± 1.7	73.1 ± 1.4 kg	180.4 ± 1.0 cm	Road, Track and Mountain Bike	Participants were categorized as Local, National (i.e., top domestic competition) or International level	13.9 ± 0.8 h	To investigate the perceptions and practices of achieving ‘race weight’ in a population of trained male cyclists. A secondary focus was to investigate the use of gym-based strength training, a possible attenuator of the side effects associated with weight reduction.	Cross-sectional self-reported survey	Survey created by research team	49% of cyclists reported being unsatisfied with their current body mass in relation to performance	Although the questionnaire employed in the present study enabled the authors to capture a wealth of data from a wider network of participants, they acknowledge that it had not been validated and was a limitation of the study. The self-reporting nature of the survey carries a risk of inaccurate data being submitted and having predetermined options for participants to select in certain questions may have imprinted some answers and influenced responses.
Sousa Fortes et al., 2017 [57] Brazil	43	Risk of ED: 21.33 ± 1.84 No risk of ED: 21.49 ± 1.75	Not reported	Not reported	Road cycling	Not reported	Risk of ED: 10.23 ± 1.12 No risk of ED: 10.26 ± 1.07 per week	The aim of this study was to compare the maximum oxygen consumption (VO2max) between road cyclists with and without risk for eating disorders.	Cross-sectional self-reported survey	Eating Attitudes Test 26 (EAT-26)	25% cyclists reported ED risk based on EAT-26 score *	A questionnaire was used to measure DEB frequency. Since responses are subjective, athletes may not have indicated total truthfulness in their responses.
Hale and Divin [61] 2011 USA	98 (M)	34 ± 4.1	Not reported	Not reported	Not reported	Amateur to professional	Not reported	To examine eating behaviours in competitive male cyclists across racing categories.	Cross-sectional self-reported survey	Eating Attitudes Test 26 (EAT-26)	Category 2 cyclists demonstrated greatest average EAT-26 score (19.5 ± 7.3) and may be at greatest risk from EDs *	None reported by the authors
Viner et al., 2015 [35] USA	10 (F and M)	Male: 42.0 ± 7.7 Female: 38.4 ± 10.3	Male: 72.4 ± 6.8 kg Female: 62.8 ± 12.2 kg	Male: 177.9 ± 4.2 cm Female: 165.4 ± 6.4 cm	Road cycling and mountain biking	Competitive	Male: 1.4 ± 0.6 Female: 0.8 ± 0.4 per day	To analyse eating behaviours that may contribute to LEA.	Cross-sectional self-reported survey	The Three-Factor Eating Questionnaire (TFEQ)—participants who scored 10 or higher were considered restrained eaters (RE) who consciously limit EI as a means of weight control	Most cyclists were identified as restricted eaters (100% road cyclists, 40% mountain bikers, 67% male and 75% female cyclists)	Small sample size
de Bruin and Oudejans 2018 [39] Netherlands	8 mixed sport (1 cyclist) (F)	Not reported	Not reported	Not reported	Not reported	International	Not reported	To explore the role of contextual body image in the development of EDs in female athletes participating in at-risk sports.	Qualitative	Interview	Increased body awareness within the elite cycling environment and thin-idealisation within the sporting context	Challenge for some participants to distinguish the different influences on their ED history as revealed by the narrative analysis In the present study, the authors combined a content analysis and narrative inquiry, which seems quite uncommon compared to other qualitative studies, and might also be taken as undesirable from a methodological point of view.
Cook and Dobbin 2022 [36] UK	36 (M)	23.1 ± 3.9 (18–30)	70.4 ± 7.1 kg	180.5 ± 6.1 cm	Road cycling	Elite (n = 8), category 1 (n = 9), category 2 (n = 19)	16.4 ± 3.2 (10–22) per week	To assess the association between sports nutrition knowledge, nutrition intake, energy availability and training characteristics with the risk of an eating disorder amongst highly trained, competitive male cyclists.	Cross-sectional self-reported survey	The Brief Eating Disorder in Athletes Questionnaire (BEDA-Q)	Four (11%) cyclists reported as being high-risk for ED Small negative correlation (r = −0.55) between SNKQ and BEDA-Q scores	The data collected in this study were self-reported by the participant due to this being conducted during a pandemic; therefore, the data may have been misreported by the participants. This is particularly important with the food dairy where participants could, either deliberately or accidentally, fail to record certain foods on the food diary. The participants may have altered their habitual diet to improve the perception of what they were eating and therefore altered their energy intake. Another limitation is the various methods to calculate each component (intake, expenditure, lean muscle mass) of the energy availability formula used in this study, as each of these may bring about a degree of error when compared to a criterion measure (e.g., indirect calorimetry, DXA). We note that the method used to estimate lean body mass has not been validated in athlete populations such as cyclists where lean mass is likely to constitute a larger proportion of total mass. Finally, the authors did not ask about the participants’ educational background, previous or current socioeconomic status or currently living arrangements—all of which might be important when considering educational resources or developing workshops to improve knowledge and energy availability.
Cook and Luke 2017 [56] USA	179 (F and M)	32.5 ± 12.9	Not reported	Not reported	Not reported	High school to professional	Not reported	To establish prevalence rates for primary and secondary EXD in a sample of cyclists. Our secondary purpose was to examine potential differences in EXD and level of competition, competition history and exercise amount.	Cross-sectional self-reported survey	Drive for Thinness subscale of the Eating Disorder Inventory-2	Low drive for thinness scores in at-risk, symptomatic, and asymptomatic secondary exercise-dependent cyclists	First, the study used a cross-sectional design and therefore cannot provide causal inferences about EXD in cyclists. Second, passive recruitment was used to obtain participants, which limits the ability for the results to be reflective of all cyclists. For example, the sample was overwhelmingly amateur-level athletes. Third, while the relatively low N is a limitation, it is within the range of previously published adequately powered cross-sectional sport and exercise psychology studies and previous EXD prevalence studies.

* When using the EAT-26 a score of 20 or greater indicated further diagnostic investigation from a qualified professional.

## Data Availability

The data and literature search protocol underpinning this paper can be requested from the corresponding author.
